# Histoplasmosis in HIV-Infected Persons, Yaoundé, Cameroon

**DOI:** 10.3201/eid2111.150278

**Published:** 2015-11

**Authors:** Christine E. Mandengue, Antoinette Ngandjio, Paul J.A. Atangana

**Affiliations:** Université des Montagnes, Bangangté, Cameroon (C.E. Mandengue);; Centre Pasteur du Cameroun, Yaoundé, Cameroon (A. Ngandjio, P.J.A. Atangana)

**Keywords:** Cameroon, HIV/AIDS and other retroviruses, tuberculosis, histoplasmosis, fungi, viruses

**To the Editor:** In HIV-infected persons in Cameroon (Central Africa), histoplasmosis is still misdiagnosed as tuberculosis because of clinical similarities (*1*,*2*). These patients are automatically given presumptive antituberculous therapy, although tuberculosis is not confirmed. The patients subsequently die of probable disseminated histoplasmosis (DH), and the fungal infection might finally be detected in postmortem tissue samples (*3*). In this context, 3 cases of DH were detected in HIV-infected patients within a 1-year period (2007–2008) in Yaoundé, Cameroon. We initiated this study to investigate the occurrence of histoplasmosis in HIV-infected patients in 4 medical centers for AIDS treatment in Yaoundé from December 2008 through December 2011.

We recruited patients with known HIV status who agreed to participate in the study. Inclusion criteria were CD4 cells <200/mm^3^, fever and cough of >2 weeks’ duration, weight loss, asthenia, and histoplasmosis-like skin manifestations (i.e., ulcerative lesions and/or umbilicated papules or nodules and/or pustules). Patients under effective antituberculous therapy or antimicrobial drugs for any skin or pulmonary infectious disease were excluded from the study. CD4 cell counts were performed in all patients. Histoplasmosis was diagnosed in sputum, bronchoalveolar fluid (BALF), and bronchial and skin biopsies by direct staining with Gomori’s methenamine silver and periodic acid Schiff stains and by culture of sputum and BALF samples on Sabouraud medium. Tuberculosis and bacterial infections were detected in sputum and BALF by using Ziehl-Neelsen and Gram staining and culture on Lowenstein-Jensen and *Streptococcus* *pyogenes* media. All laboratory examinations were performed at the Centre Pasteur du Cameroun in Yaoundé. Data were collected on an anonymous questionnaire. Means (and SDs) were calculated for quantitative variables, and frequencies were calculated for qualitative variables. The National Ethics Committee, the Ministry of Health of Cameroon, and the medical centers where the study took place approved the study. Patients approved and signed the informed consent form at the time of recruitment.

Our study comprised 56 patients. *Histoplasma capsulatum* was detected in 7 (13%) patients on 6 of 7 skin biopsies and 1 of 3 bronchial biopsies. The median CD4 cell count of *H. capsulatum*–positive patients was 40 cells/mm^3^. Similarly, some authors have reported diagnosis of severe DH by using direct staining of skin samples (*4*); in low-income countries, skin involvement is the main presentation of DH because of limited laboratory facilities and/or late diagnosis. In Cameroon until recently, all DH cases in HIV-infected persons were diagnosed by skin biopsy or by chance on peripheral blood smear, thus revealing AIDS at the terminal stage (*3*,*5*). We did not detect *H. capsulatum* infection in sputum or BALF. These results are congruent with findings in Abidjan, Côte d’Ivoire, in 1999 (*6*). African histoplasmosis was not detected in any sample; although this type is endemic to areas with high rates of HIV infection, it is infrequently associated with AIDS patients (*7*).

We detected *Mycobacterium tuberculosis* in 18 (32%) patients and *Candida albicans* in 14 (25%) patients; 3 (0.5%) patients were co-infected with *M. tuberculosis* and *C. albicans*. *M. tuberculosis* was detected in sputum of 9 (21%) of 42 patients and in BALF of 9 (53%) of 17 patients; we detected *C. albicans* in sputum of 13 (31%) patients. Our detection of *M. tuberculosis* in 32% of patients confirms tuberculosis as the main AIDS-defining illness in Cameroon. We did not find tuberculosis and histoplasmosis co-infection, even though it occurs frequently in low-income countries (*1*,*8*).

The limitation in our study was the unavailability of validated sensitive and specific tools for diagnosing histoplasmosis in Cameroon (e.g., detection of the* H. capsulatum* circulating antigen in body fluid using an enzyme immunoassay) (*9*). Thus, using direct staining methods and culture of biopsies and body fluid samples could possibly lead to false-negative results.

Our detection of *H. capsulatum* in 13% of the HIV-infected patients in this study suggests that histoplasmosis is an unknown public health problem in Cameroon that is misdiagnosed as tuberculosis. Accounting for the endemicity of tuberculosis, which is the main HIV-defining illness in Cameroon, and the fatal outcome of DH in HIV-infected patients, practitioners need a high index of awareness to differentiate between tuberculosis and histoplasmosis. A recent report showed major clinical and biologic factors discriminating between these infections (*10*). Knowing these factors may lead practitioners to early diagnosis and treatment of histoplasmosis and in turn reduce the death rate among HIV-infected patients.
